# Evaluating Cardiovascular Benefits of Glucagon-Like Peptide-1 Receptor Agonists (GLP-1 RAs) in Type 2 Diabetes Mellitus: A Systematic Review

**DOI:** 10.7759/cureus.66697

**Published:** 2024-08-12

**Authors:** Chithra Sreenivasan, Aneri Parikh, Aida J Francis, Tatchaya Kanthajan, Manorama Pandey, Osamah AlQassab, Tuheen Sankar Nath

**Affiliations:** 1 Internal Medicine, California Institute of Behavioral Neurosciences & Psychology, Fairfield, USA; 2 Surgical Oncology, California Institute of Behavioral Neurosciences & Psychology, Fairfield, USA

**Keywords:** cardiovascular adverse effects, cardiovascular outcomes, glp-1 ra, glucagon-like peptide-1 receptor agonist, type 2 diabetes mellitus

## Abstract

Cardiovascular risks and complications remain elevated in patients with type 2 diabetes even after appropriate control of contributing factors like glycemic control, hypertension, and lipid profile. More efficient methods are needed to address this issue in type 2 diabetics. Newer drugs like glucagon-like peptide-1 receptor agonists (GLP-1 RAs) have shown a cardioprotective effect in addition to glycemic control. This systematic review aims to study the latest literature findings on the cardiovascular effects of GLP-1 RAs in patients with type 2 diabetes. We used PubMed, Google Scholar, Science Direct, and Biomed Central databases for our data collection. Our review adheres to the 2020 Preferred Reporting Items for Systematic Reviews and Meta-analysis (PRISMA) guidelines. The outcomes evaluated in the review include major adverse cardiovascular events (MACE), heart failure, stroke, all-cause mortality, and effects on cardiovascular risk factors. After careful inspection and quality check, we included 14 articles in the systematic review. GLP-1 RAs were associated with a significant reduction in cardiovascular mortality, all-cause mortality, nonfatal myocardial infarction (MI), and nonfatal stroke, especially in patients with existing cardiovascular risk factors. However, more evidence is required to determine if these benefits extend to those without such risk factors. Limited data suggest that GLP-1 RAs might have a protective effect on arrhythmias, but this area needs further investigation. Despite their potential, several barriers hinder the widespread use of GLP-1 RAs. In conclusion, GLP-1 RAs significantly reduce cardiovascular mortality, all-cause mortality, nonfatal MI, and stroke, with minor effects on hospitalization due to heart failure. Benefits are greater in patients with cardiovascular risk factors. A comprehensive, multilevel approach to policy development and implementation is necessary to optimize the use of these medications in eligible populations.

## Introduction and background

One of the most significant public health concerns of the twenty-first century is type 2 diabetes. By 2025, according to rising prevalence rates, approximately 570 million cases of the illness worldwide are projected, which will pose an economic and social burden globally [1,2]. Individuals with type 2 diabetes face a heightened risk of developing cardiovascular conditions, such as myocardial infarction (MI), heart failure (HF), stroke, peripheral artery disease, and cardiovascular death. Aggressive glucose-lowering treatments have not been convincingly effective in lowering cardiovascular morbidity and mortality in diabetes patients at high cardiovascular risk. Addressing cardiovascular complications of type 2 diabetes remains an unmet need, with its associated expenditures related to medications, hospitalizations, and interventions posing a significant financial burden [3]. Substantial evidence suggests that the novel glucose-lowering agents, particularly glucagon-like peptide-1 receptor agonists (GLP-1 RAs), reduce the incidence of significant cardiovascular events in those who have type 2 diabetes [4-6].

GLP-1 RAs

Glucagon-like peptide-1 (GLP-1) is a short peptide hormone produced by gastrointestinal L cells in response to nutrition consumption. Various human tissues, including pancreatic islets, stomach, brain, endothelial cells, lungs, kidneys, smooth muscle cells, and particular atrial and ventricular cardiomyocytes, exhibit GLP-1 expression. GLP-1 binds to the GLP-1 receptor and causes incretin actions such as glucose-dependent insulin production from pancreatic β cells, suppression of glucagon release from pancreatic α cells, and delayed gastric emptying. Together, these effects reduce blood glucose levels and promote postprandial glucose metabolism. Additionally, GLP-1 stimulates glucagon-like peptide-1 receptor (GLP-1R)-expressing hypothalamus neurons, which promote satiety and weight reduction. GLP-1 has a half-life of only a few minutes due to its cleavage by the ubiquitously expressed enzyme dipeptidyl peptidase-4 (DPP4). GLP-1 RAs are peptides identical to human GLP-1, cannot be cleaved by DPP4, and are complete agonists of GLP-1R. Liraglutide, semaglutide, and dulaglutide are examples of GLP-1 RA that were made using human GLP-1 as a basis. They lower glycated hemoglobin (HbA1c) by 0.8-1.5%, significantly reducing weight. GLP-1 RAs not only reduce postprandial glucose excursions but also lower fasting plasma glucose [4-6].

Cardiovascular effects of GLP-1 RAs

Data from large-scale cardiovascular outcome trials (CVOTs) have shown a significant and persistent decrease in atherothrombotic events, particularly among patients with preexisting atherosclerotic cardiovascular disease [7-12]. All available GLP-1 RAs have an impact on cardiovascular risk factors. GLP-1 RA reduces systolic blood pressure by 2-6 mmHg, linked to reduced cardiovascular events. Previous findings from hypertension studies indicate that lowering blood pressure leads to a considerable reduction in major adverse cardiovascular events (MACE). Regarding lipids, GLP-1 RA lowers total cholesterol, low-density lipoprotein (LDL), and triglycerides, indicating a possible positive impact. Various experimental data have shown that GLP-1 and GLP-1 RA reduce the development and progression of atherosclerotic lesions by resulting in more stabilized and less vulnerable plaques, most likely due to antiatherogenic and anti-inflammatory effects in endothelial cells, monocytes, macrophages, and vascular smooth muscle cells that express GLP-1R [4-6].

The placebo-controlled cardiovascular outcome trials examined MACE, nonfatal MI, stroke, or all-cause mortality. A decreased incidence of MACE was observed in four of the studies, namely LEADER for liraglutide (Liraglutide Effect and Action in Diabetes: Evaluation of Cardiovascular Outcome Results), SUSTAIN 6 for weekly semaglutide (Trial to Evaluate Cardiovascular and Other Long-term Outcomes with Semaglutide in Subjects With Type 2 Diabetes), Harmony Outcomes for albiglutide, and REWIND for dulaglutide (Researching Cardiovascular Events With a Weekly Incretin in Diabetes). These findings have led to the prioritizing of novel anti-hyperglycemic drugs for secondary prevention of unfavorable cardiovascular consequences in individuals with type 2 diabetes [8-12].

Despite the robust evidence supporting their use, integrating GLP-1 RAs into routine clinical practice remains suboptimal. Barriers such as cost, patient adherence, and physician familiarity with these therapies persist [13]. Overcoming these challenges through policy and education is crucial to enhancing the utilization of GLP-1 RAs, ultimately improving cardiovascular outcomes in the diabetic population.

In this systematic review, we have included clinical trials, meta-analyses, and systematic reviews published from 2020 to 2024. We aim to gather the latest findings from the literature on cardiovascular risks and benefits assessment in type 2 diabetes patients on GLP-1 RAs. We also aim to analyze the underuse of GLP-1 RAs in clinical practice despite the increasing evidence demonstrating their efficacy. Outcomes evaluated in the review include MACE, heart failure, stroke, all-cause mortality, and effects on cardiovascular risk factors.

## Review

Methods

Our review adheres to the 2020 Preferred Reporting Items for Systematic Reviews and Meta-Analysis (PRISMA) guidelines [14].

Database and Search Methodology

We performed our data search from April 22, 2024, to April 30, 2024. We used PubMed, Google Scholar, Science Direct, and Biomed Central databases for our data collection. Additionally, grey literature, including unpublished studies, reports, and theses, was used as a source. We searched for studies related to cardiovascular outcomes in type 2 diabetic patients on GLP-1 or dual GLP-1/gastric inhibitory polypeptide (GIP). Table 1 lists the search strategy, keywords, and medical subject headings (MeSH) used for data collection.

**Table 1:** Search strategy GLP-1 RA: glucagon-like peptide-1 receptor agonist

Database	Keywords	Search strategy	No. of records before applying filters	Filters	No. of records after applying filters
PubMed	Type 2 diabetes mellitus, glucagon-like peptide-1 receptor agonist, GLP-1 RA, cardiovascular outcomes, cardiovascular adverse effects	("Cardiovascular Diseases/drug therapy" [Majr] OR "Cardiovascular Diseases/pathology" [Majr] OR "Cardiovascular Diseases/physiopathology" [Majr] OR "Cardiovascular Diseases/therapy" [Majr]) AND ("Glucagon-Like Peptide-1 Receptor Agonists/administration and dosage" [Majr] OR "Glucagon-Like Peptide-1 Receptor Agonists/adverse effects" [Majr] OR "Glucagon-Like Peptide-1 Receptor Agonists/therapeutic use" [Majr] OR "Glucagon-Like Peptide-1 Receptor Agonists/toxicity" [Majr]) OR ("Liraglutide/administration and dosage" [Majr] OR "Liraglutide/adverse effects" [Majr] OR "Liraglutide/therapeutic use" [Majr] OR "Liraglutide/toxicity" [Majr]) OR ("Exenatide/adverse effects" [Majr] OR "Exenatide/agonists" [Majr] OR "Exenatide/analogs and derivatives "[Majr] OR "Exenatide/therapeutic use" [Majr] OR "Exenatide/toxicity" [Majr]) OR "tirzepatide" [Majr] AND ("Diabetes Mellitus, Type 2/complications" [Majr] OR "Diabetes Mellitus, Type 2/drug therapy" [Majr] OR "Diabetes Mellitus, Type 2/mortality" [Majr] OR "Diabetes Mellitus, Type 2/pathology" [Majr] OR "Diabetes Mellitus, Type 2/physiopathology" [Majr] OR "Diabetes Mellitus, Type 2/prevention and control" [Majr] OR "Diabetes Mellitus, Type 2/therapy" [Majr])	523	Free full-text, English, systematic review, meta-analysis, clinical trial, 2020-2024	46
Google Scholar	Type 2 diabetes mellitus, glucagon-like peptide-1 receptor agonist, GLP-1 RA, cardiovascular outcomes, cardiovascular adverse effects	(GLP-1 RA AND "diabetes mellitus type 2") and (Cardiovascular risk and outcome)	1,920	2020-2024, English	1,400
ScienceDirect	Type 2 diabetes mellitus, glucagon-like peptide-1 receptor agonist, GLP-1 RA, cardiovascular outcomes, cardiovascular adverse effects	(GLP-1 RA AND "diabetes mellitus type 2") and (Cardiovascular risk and outcome)	76	2020-2024	40
MedRxiv	Type 2 diabetes mellitus, glucagon-like peptide-1 receptor agonist, GLP-1 RA, cardiovascular outcomes, cardiovascular adverse effects	(GLP-1 RA AND "diabetes mellitus type 2") and (Cardiovascular risk and outcome)	620	2020-2024	12
BioMed Central	Type 2 diabetes mellitus, glucagon-like peptide-1 receptor agonist, GLP-1 RA, cardiovascular outcomes, cardiovascular adverse effects	GLP-1 RA AND “type 2 diabetes” AND cardiovascular outcomes	196	2020-2024	70

Inclusion and Exclusion Criteria

We included clinical trials, systematic reviews, and meta-analyses published in English between 2020 and 2024. The studies had to involve adults aged 18 and above diagnosed with type 2 diabetes mellitus and examine the cardiovascular effects of GLP-1 RAs or dual GLP-1/GIP therapies. We focused on studies reporting MACE, heart failure, stroke, all-cause mortality, and cardiovascular risk factors. We considered only articles available as free full-text. We excluded animal studies, in vitro studies, case reports, editorials, commentaries, studies involving type 1 diabetes patients, children, or patients with renal failure, and articles published before 2020 or in languages other than English.

Data Extraction

We aimed to include only high-quality studies in this systematic review. Two authors independently screened and extracted data based on the established inclusion and exclusion criteria. We resolved differences in opinions through consensus, seeking a third reviewer's opinion as needed.

Search Results

**Figure 1:**
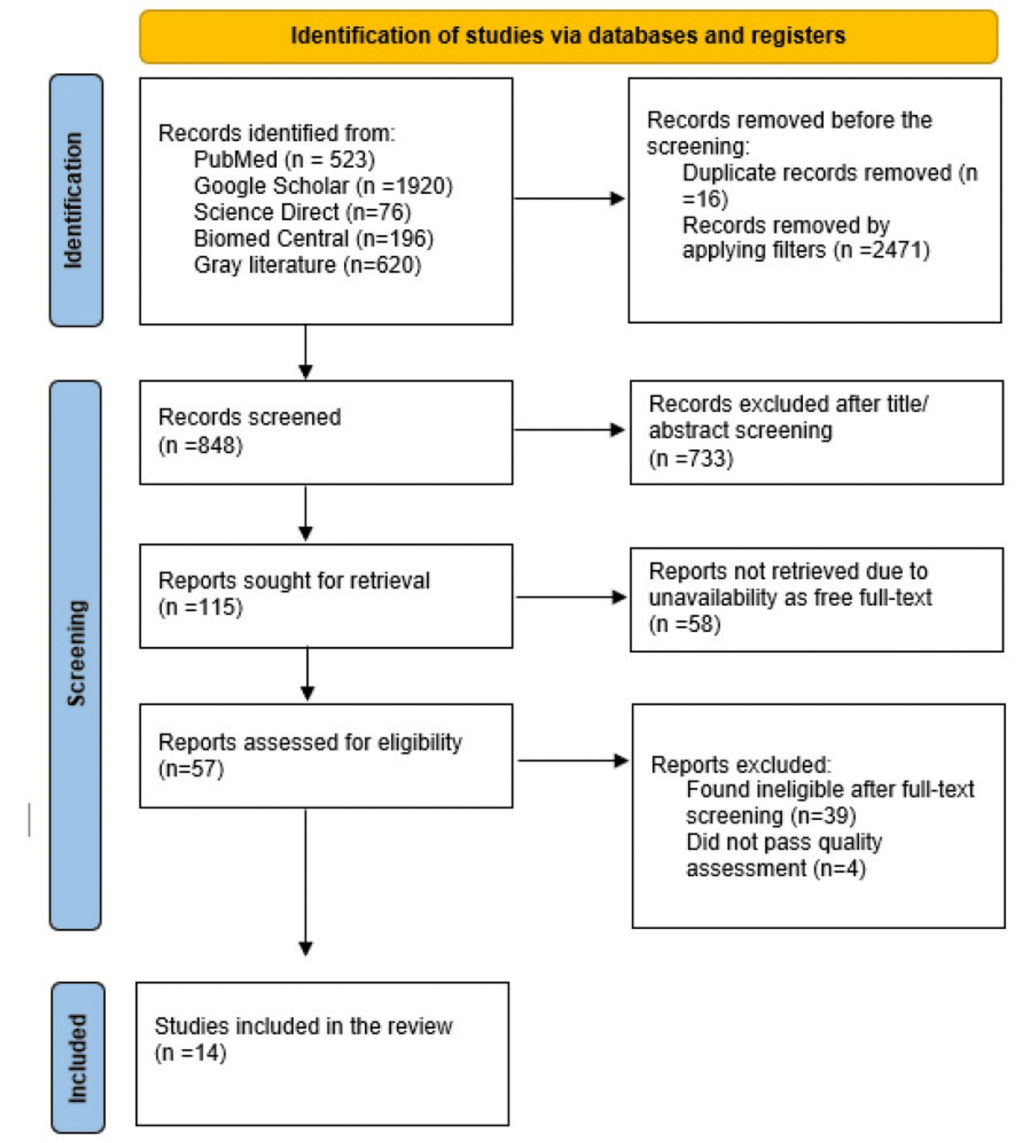
PRISMA flow diagram PRISMA: Preferred Reporting Items for Systematic Reviews and Meta-Analysis

The literature search conducted through PubMed, Google Scholar, Science Direct, Biomed Central, and grey literature sources via MedRxiv yielded 3,335 articles. After applying filters and removing duplicates, we screened the remaining articles by title and abstract. Two independent reviewers conducted this screening, resulting in the exclusion of 733 irrelevant articles. In addition, we excluded 58 articles due to the unavailability of free full text. After thoroughly reviewing the full-text versions, we excluded 39 more articles. Ultimately, we assessed the quality of 18 articles and included 14 high-quality studies in our review. Refer to Figure 1 for the detailed PRISMA flow chart [14].

Critical Appraisal

For quality appraisal, we used the Cochrane risk of bias assessment for randomized controlled trials (RoB 2), the Newcastle-Ottawa Scale (NOS) for observational trials, and the Assessment of Multiple Systematic Reviews (AMSTAR 2) checklist for systematic reviews and meta-analysis. We included only studies fulfilling the criteria of quality defined by AMSTAR 2, NOS, and RoB2 in the review [15-17]. Tables 2-5 show the detailed quality assessment of the included studies. Table 6 shows the study characteristics of the final 14 articles in the review.

**Table 2:** Critical appraisal of included studies AMSTAR 2: Assessment of Multiple Systematic Reviews; RoB 2: Cochrane risk of bias assessment for randomized controlled trials

Author	Type of study	Quality assessment tool	Quality score
Scheen et al. [18]	Systematic review and meta-analysis	AMSTAR 2	High-quality review
Rahman et al. [19]	Systematic review and meta-analysis	AMSTAR 2	High-quality review
Qin et al. [20]	Meta-analysis	AMSTAR 2	High-quality review
Parab et al. [21]	Systematic review	AMSTAR 2	High-quality review
Nogueira et al. [22]	Systematic review and meta-analysis	AMSTAR 2	High-quality review
Kunutsor et al. [23]	Systematic review and meta-analysis	AMSTAR 2	High-quality review
Giugliano et al. [24]	Meta-analysis	AMSTAR 2	High-quality review
Evans et al. [25]	Systematic review and meta-analysis	AMSTAR 2	High-quality review
Thein et al. [26]	Prospective cohort	Newcastle-Ottawa Scale	High quality
Piccini et al. [27]	Retrospective cohort	Newcastle-Ottawa Scale	High quality
Longato et al. [28]	Retrospective cohort	Newcastle-Ottawa Scale	High quality
Huang et al. [29]	Retrospective cohort	Newcastle-Ottawa Scale	High quality
Tuttolomondo et al. [30]	Randomized controlled trial	RoB 2	Low risk of bias
Zhang et al. [31]	Randomized controlled trial	RoB 2	Low risk of bias

**Table 3:** AMSTAR 2 checklist for systematic reviews and meta-analysis AMSTAR 2: Assessment of Multiple Systematic Reviews; RCT: randomized controlled trials; NRSI: non-randomized studies of interventions

Questions	Scheen et al. [18]	Rahman et al. [19]	Qin et al. [20]	Parab et al. [21]	Nogueira et al. [22]	Kunutsor et al. [23]	Giugliano et al. [24]	Evans et al. [25]
Did the research questions and inclusion criteria for the review include the components of PICO?	Yes	Yes	Yes	Yes	Yes	Yes	Yes	Yes
Did the report of the review contain an explicit statement that the review methods were established prior to the conduct of the review, and did the report justify any significant deviations from the protocol?	Yes	Yes	Yes	Yes	Yes	Yes	Yes	Yes
Did the review authors explain their selection of the study designs for inclusion in the review?	Yes	Yes	Yes	Yes	Yes	Yes	Yes	Yes
Did the review authors use a comprehensive literature search strategy?	Yes	Yes	Yes	Yes	Yes	Yes	Yes	Yes
Did the review authors perform study selection in duplicate?	Yes	Yes	Yes	Yes	Yes	Yes	Yes	Yes
Did the review authors perform data extraction in duplicate?	Yes	Yes	Yes	Yes	Yes	Yes	Yes	Yes
Did the review authors provide a list of excluded studies and justify the exclusions?	Yes	Yes	Yes	Yes	Yes	Yes	Yes	Yes
Did the review authors describe the included studies in adequate detail?	Yes	Yes	Yes	Yes	Yes	Yes	Yes	Yes
Did the review authors use a satisfactory technique for assessing the risk of bias (RoB) in individual studies that were included in the review?	RCT - Yes NRSI - Yes	RCT - Yes NRSI - Yes	RCT - Yes NRSI - Yes	RCT - 0 NRSI - 0	RCT - Yes NRSI - Yes	RCT - Yes NRSI - Yes	RCT - Yes NRSI - Yes	RCT - Yes NRSI - Yes
Did the review authors report on the sources of funding for the studies included in the review?	Yes	Yes	Yes	Yes	Yes	Yes	Yes	Yes
If meta-analysis was performed, did the review authors use appropriate methods for statistical combination of results?	RCT - Yes NRSI - Yes	RCT - Yes NRSI - Yes	RCT - Yes NRSI - Yes	RCT - 0 NRSI - 0	RCT - Yes NRSI - Yes	RCT - Yes NRSI - Yes	RCT - Yes NRSI - Yes	RCT - Yes NRSI - Yes
If meta-analysis was performed, did the review authors use appropriate methods for statistical combination of results?	Yes	Yes	Yes	Yes	Yes	Yes	Yes	Yes
If meta-analysis was performed, did the review authors assess the potential impact of RoB in individual studies on the results of the meta-analysis or other evidence synthesis?	Yes	Yes	Yes	Yes	Yes	Yes	Yes	Yes
Did the review authors account for RoB in individual studies when interpreting/ discussing the results of the review?	Yes	Yes	Yes	Yes	Yes	Yes	Yes	Yes
Did the review authors provide a satisfactory explanation for and discussion of any heterogeneity observed in the results of the review?	Yes	Yes	Yes	Yes	Yes	Yes	Yes	Yes
If they performed quantitative synthesis, did the review authors carry out an adequate investigation of publication bias (small study bias) and discuss its likely impact on the results of the review?	Yes	Yes	Yes	Yes	Yes	Yes	Yes	Yes

**Table 4:** Newcastle-Ottawa Scale for observational trials

	Selection				Comparability		Outcome			
Study	Representative of the exposed cohort	Selection of external control	Ascertainment of exposure	Outcome of interest not present at the start of the study	Main factor	Additional factor	Assessment of outcomes	Sufficient follow-up time	Adequacy of follow-up	Total (9/9)
Thein et al. [26]	*	*	*	*	*	*	*	*	*	9/9
Piccini et al. [27]	*	*	*	0	*	*	*	*	*	8/9
Longato et al. [28]	*	*	*	*	*	*	*	*	*	9/9
Huang et al. [29]	*	*	*	*	*	0	*	*	*	8/9

**Table 5:** Cochrane risk of bias assessment for randomized controlled trials

Study	Randomization process	Deviations from the intended interventions	Missing outcome data	Measurement of the outcome	Selection of the reported result
Tuttolomondo et al. [30]	Low risk	Low risk	Low risk	Low risk	Low risk
Zhang et al. [31]	Low risk	Low risk	Low risk	Low risk	Low risk

**Table 6:** Characteristics of included studies T2DM: type 2 diabetes mellitus; CVD: cardiovascular disease; GLP-1 RA: glucagon-like peptide-1 receptor agonists; SGLT-2: sodium-glucose cotransporter-2; RCTs: randomized controlled trials; CVOTs: cardiovascular outcome trials; CIMT: carotid intima-media thickness; RR: relative risk; HR: hazard ratio; CI: confidence interval; MI: myocardial infarction; MACE: major adverse cardiovascular events; ASCVD: atherosclerotic cardiovascular disease; HbA1c: hemoglobin A1c; DPP4: dipeptidyl peptidase-4; DPP4i: DPP4 inhibitor; SU: sulfonylurea; SGLT-2i: SGLT-2 inhibitor; GLP-1A: glucagon-like peptide-1 agonist; 3P-MACE: 3-point MACE

Author	Population characteristics	Outcomes	Limitations
Scheen et al. [18]	Nine cohorts from seven studies collected from international literature between 2019 and 2022	In seven out of nine cohorts, the usage of GLP-1 RAs in patients with ASCVD was marginally less than in individuals without ASCVD (odds ratio: 0.80; 95% CI: 0.79-0.81).	Most trials reported a relatively low percentage of individuals treated with GLP-1 RAs.
Rahman et al. [19]	64,452 patients received either liraglutide (three studies) or exenatide (two studies)	Substantially low risk of MACE (0.72 (95% CI: 0.65 - 0.93; I^2^ = 68%)) extended MACE (0.93 (95% CI: 0.89 - 0.98; I^2^ = 29%)), all-cause mortality (0.82 (95% CI: 0.76 - 0.88; I^2^ = 0%)), and CV mortality (0.75 (95% CI: 0.65 - 0.85; I^2 ^= 38%)) were linked to GLP-1 RA treatment.	The smaller number of eligible studies is a limitation because of the numerous and specific inclusion criteria. The meta-analysis for heart failure included only two studies, which limited the observations.
Qin et al. [20]	Six multinational, double-blind, randomized, placebo-controlled trials that enrolled a total of 52,821 patients with T2DM	GLP-1 RAs decreased the risk of mortality from cardiovascular causes (RR: 0.90; 95% CI: 0.83-0.97; P = 0.004) and fatal or nonfatal stroke (RR: 0.85; 95% CI: 0.77-0.94; P = 0.001) when compared to placebo controls.	Patients who received different drugs of the same class can introduce bias in the meta-analysis results. Publication bias is also a limitation of this meta-analysis.
Parab et al. [21]	Systematic review based on 14 articles	GLP-1 RAs show a substantial 12-14% decrease in the 3-point composite MACE outcome.	Individual drugs belonging to the class may have specific effects not explored in the study.
Nogueira et al. [22]	157 RCTs included 267,508 patients spread across 176 active arms, with an average follow-up time of 1.46 years. GLP-1A decreased the relative risk of nonfatal cardiovascular events (HR: 0.79 (95% CI: 0.67-0.94))	GLP-1A demonstrated reduced relative risks of nonfatal cardiovascular events (HR: 0.79 (95% CI: 0.67-0.94)).	The study's results are based on therapies in Brazil, which limits their extrapolation to other countries, given that drug prices can vary significantly. The incidence rates of several outcomes were based on small cohorts, leading to uncertainty regarding the statistical power to detect less frequent clinical outcomes.
Kunutsor et al. [23]	20 unique CVOTs (six SGLT-2is, nine GLP-1 RAs, five DPP4is), based on 169 513 participants with T2D	GLP-1 RAs consistently decreased the risk of several macrovascular and microvascular complications. The HR (95% CIs) for three-point major adverse cardiovascular events was 0.85 (0.79-0.92).	Certain results had a low occurrence rate as they were derived from individual studies. All outcomes were based on a pooled analysis of less than ten studies, which restricted the depth of evaluation and may have introduced biases.
Giugliano et al. [24]	Eight CVOTs and 60,080 patients (72.4% with established cardiovascular disease)	GLP-1 RA reduced major cardiovascular events (MACE) by 14% (HR: 0.86; 95% CI: 0.79-0.94; P = 0.006).	This is a meta-analysis of trial data, which is inferior to patient-level meta-analysis. The study used aggregate data, so patient subgroups (with and without cardiovascular disease) could not be investigated. Differences in gender were not considered, and the possible superiority of a drug within the GLP1-RA class was also not investigated.
Evans et al. [25]	22 studies involving over 200,000 participants were pooled	Treatment with GLP-1 RA was associated with a more significant benefit on composite cardiovascular outcomes (HR: 0.77; 95% CI: 0.69-0.87), myocardial infarction (HR: 0.82; 95% CI: 0.69-0.97), stroke (HR: 0.83; 95% CI: 0.74-0.93), cardiovascular mortality (HR: 0.76; 95% CI: 0.68-0.85) and all-cause mortality (HR: 0.65; 95% CI: 0.48-0.90).	Differences in the patient population, research designs, comparators, follow-up periods, and end goals can cause heterogeneity in studies, which can limit the interpretation. The difference in body weight of the patients can contribute to the findings observed but was not accounted for in the study. The duration of diabetes, HbA1c levels, and coexisting metabolic conditions, which are important predictors of cardiovascular risk, were also not adequately accounted for in the study.
Thein et al. [26]	46,986 T2D patients with an average age of 61 years, of which 59% were male, previously on metformin monotherapy, who initiated an additional glucose-lowering agent (GLP-1 RA, SGLT-2i, DPP4i, SU, or insulin) in the period 2010-2016	When compared to add-on DPP4is, adding a GLP-1 RA or SGLT-2i to metformin therapy was linked with a similar risk of HF hospitalization and mortality, alongside a reduced risk of MACE for GLP-1 RA (HR of 0.82 (0.69-0.97)). In contrast, starting therapy with SU and insulin was related to a greater incidence of MACE (1.22 (1.03-1.49) and 1.23 (1.07-1.47) respectively). Insulin has also been linked to an increased risk of all-cause death (2.33 (2.08-2.61)) and hospitalization (1.54 (1.25-1.91)) from heart failure.	The lack of information on clinical variables, including blood glucose, kidney function, body mass index, smoking status, and blood pressure, raises the risk of confounding. The study may have considered patients with stable vascular disease as a moderate-risk group.
Piccini et al. [27]	Retrospective cohort study of 550 people with type 2 diabetes (395 in primary CV prevention, 155 in secondary CV prevention) conducted at a single facility following the first prescription of a GLP-1 RA from 2009 to 2019	For both individuals with and without a history of cardiovascular events, discontinuing GLP-1 RA treatment was linked to an elevated risk of major cardiovascular events ((HR 2.71; 95% CI: 1.46-5.01; P = 0.002) and (HR 3.40; 95% CI: 1.82-6.32; P < 0.001) respectively).	The study's retrospective design could have introduced selection bias and measurement errors. Additionally, the absence of a control group and patients' self-reported treatment adherence pose significant limitations. Furthermore, the exclusive focus on patients from a single tertiary center limits the generalizability of the findings.
Longato et al. [28]	Two matched cohorts of 2,807 DPP4i and 2,807 GLP-1 RA initiators were followed for an average of eighteen months	GLP-1 RA treatment resulted in a decreased risk of 3P-MACE compared to DPP4i (23.5 vs. 34.9 occurrences per 1000 person-years; HR: 0.67; 95% CI: 0.53-0.86; P = 0.002). GLP-1 RA initiators had decreased rates of myocardial infarction (HR: 0.67; 95% CI: 0.50-0.91; P = 0.011) and all-cause mortality (HR: 0.58; 95% CI: 0.35-0.96; P = 0.034).	There were differences in clinical characteristics between the patients included in the study. The study did not consider factors like compliance, lifestyle, or socioeconomic strata.
Huang et al. [29]	4,600 patients receiving basal insulin were matched with 1057 individuals receiving liraglutide	The liraglutide group had a decreased risk of a composite CVD outcome (HR: 0.65; 95% CI: 0.50-0.85; P < 0.01), all-cause death (HR: 0.40; 95% CI: 0.28-0.59; P < 0.0001), and nonfatal stroke (HR: 0.54; 95% CI: 0.34-0.87; P = 0.01).	Due to the lack of laboratory data in the health and welfare database, factors like blood pressure and lipid profile could not be taken into account. Data on the body mass index and patient adherence to medication regimen were also not available. Lastly, only patients with high cardiovascular risk were included, and findings cannot be generalized to all diabetic patients.
Tuttolomondo et al. [30]	A single-center, nine-month randomized trial evaluated the effectiveness of antidiabetic conventional medication with dulaglutide versus traditional antidiabetic treatment alone in 124 individuals with type 2 diabetes	Individuals with type 2 diabetes who received 1.5 mg of subcutaneous dulaglutide in addition to conventional therapy daily fared better than those who received standard care alone regarding metabolic effects and vascular health indicators such as arterial stiffness and endothelial function.	The study measured cardiovascular risk markers like blood pressure and lipid panel as outcomes rather than cardiovascular events.
Zhang et al. [31]	66 patients with T2DM were randomized to receive either twice-daily exenatide or aspartate 70/30 insulin for 52 weeks	Exenatide substantially reduced CIMT from baseline compared to insulin after 52 weeks, having a mean difference of -0.14 mm (95% confidence interval: -0.25, -0.02; P = 0.016). Over 52 weeks, the exenatide group lost considerable weight and body mass index. At weeks 16 and 40, exenatide significantly lowered total and low-density lipoprotein cholesterol levels compared to insulin. Correlation analysis revealed that CIMT was positively connected with low-density lipoprotein cholesterol.	The lack of statistical power to evaluate the effect of exenatide on some metabolic outcomes and the lack of comparison of cardiovascular outcomes associated with exenatide to those of insulin are limitations. Study participants were free to use metformin, and it is unclear whether the combination of metformin and exenatide affects the outcomes.

Discussion

MACE

GLP-1 RAs and their various beneficial effects have been a topic of interest ever since their introduction in 2005, and various studies have been conducted on them [7-12]. While the standard MACE definition includes cardiovascular death, nonfatal MI, and nonfatal stroke, some studies expand this definition to include other relevant cardiovascular outcomes. This broader definition helps capture the full cardiovascular benefits associated with GLP-1 RAs. A systematic review and meta-analysis of the eight significant CVOTs between GLP-1 RA and insulin was done by Evans et al. in 2023. Their studies showed that GLP-1 RA showed a significantly better cardiovascular outcome when compared to basal insulin (hazard ratio (HR): 0.62; 95% confidence interval (CI): 0.48-0.79; I^2 ^= 82%) [25]. The pre-existing cardiovascular risk factors of the populations included in their studies may have influenced the extent of these benefits.

Giugliano et al. also reinforced the cardioprotective effects of GLP-1 RAs in their meta-analysis of the eight CVOTs in 60,080 patients in 2021. They found that the risk of MACE was reduced by 14% compared to placebo and that this effect was more pronounced in people with pre-existing cardiovascular risk factors than in people without (16% and 6%, respectively) [24]. Huang et al. confirmed the clinical outcomes of GLP-1 RAs observed in the LEADER trial through their retrospective cohort study [29]. They proved that the reduction in MACE and cardiovascular risk factors observed in clinical trials applied to a broader population group in the real world.

Studies also compared the anti-atherosclerotic effect of GLP-1 RA with other novel anti-hyperglycemic agents. Longato et al., in their retrospective study comparing patients on GLP-1 RA and DDP4 inhibitor (DPP4i), found that the former had a better cardiovascular outcome when compared to the latter, irrespective of the sex and pre-existing cardiovascular risk factors [28]. Their study favored the hypothesis that the anti-atherosclerotic protective effect could also apply to patient populations without pre-existing atherosclerotic cardiovascular diseases. Piccini et al. further outlined this finding in their retrospective cohort study. They observed that GLP-1 RAs enabled a long-lasting decrease in HbA1c over time. This improvement in glycemic control with a longer duration of treatment with glucagon-like peptides (GLPs) could be a potential mediator of cardiovascular benefits, responsible for up to 82% of the total reduction in atherosclerotic events, even in those without a history of cardiovascular diseases [27].

Heart Failure

Giugliano et al. also found that GLP-1 RAs decreased the risk of hospitalization due to heart failure by 10% (HR: 0.90; 95% CI: 0.83-0.98; P = 0.023) [24]. Their observation was limited because none of the studies included in their analysis considered heart failure a primary outcome. Also, this observation could have been due to the overall atherosclerotic risk reduction benefits of the class of drugs. Heart failure risk reduction was also observed in a few other studies including Evans et al. (HR: 0.57; 95% CI: 0.35-0.92), Kunutsor et al. (pooled HR (95% CI) of 0.92 (0.84-1.00)) and Thein et al. in 2020, who found that the risk was increased in patients on insulin, though there was no significant difference among the various second-line agents used [23, 25-26]. Further comprehensive studies are necessary to establish definitive conclusions regarding the benefits of GLP-1 RAs on heart failure.

Stroke

GLP-1 RAs have demonstrated a significant capacity to reduce stroke events compared to other treatment modalities. Kunutsor et al. found a substantial reduction in the risk of overall stroke (HR: 0.85; 95% CI: 0.77-0.93), fatal stroke (HR 0.72; 95% CI: 0.52-1.00), and nonfatal stroke (HR: 0.81; 95% CI: 0.71-0.93). These findings indicate that factors beyond glycemic control, such as improvements in blood pressure, lipid levels, and weight management, may contribute to the benefits of GLP-1 RAs in reducing stroke. Additionally, the anti-inflammatory properties of GLP-1 RAs and their ability to reduce oxidative stress may be crucial in lowering the risk of stroke [23]. Supporting these findings, Evans et al. showed that GLP-1 RAs reduced the incidence of stroke more significantly than insulin, with a hazard ratio (HR) (95% CI) of 0.50 (0.40-0.63). The study highlighted a 16% reduction in the risk of nonfatal stroke, emphasizing the superior efficacy of GLP-1 RAs in stroke prevention compared to traditional insulin therapy [25].

Furthermore, Huang et al. discovered that patients treated with liraglutide, a specific GLP-1 RA, had a significantly lower risk of stroke (HR: 0.54; 95% CI: 0.34-0.87). This study also highlighted the broader cardiovascular benefits of liraglutide, including a reduced risk of a composite cardiovascular outcome, lower all-cause mortality, and reduced healthcare costs related to cardiovascular events [29]. These studies suggest that GLP-1 RAs provide substantial cardiovascular benefits beyond glycemic control. These benefits include improvements in blood pressure, lipid profiles, weight management, anti-inflammatory effects, and reduction of oxidative stress. These multifactorial advantages position GLP-1 RAs as a superior treatment option for reducing stroke and other cardiovascular events in type 2 diabetes patients.

Other Effects and All-Cause Mortality

Evans et al. observed that GLP-1 RAs reduced cardiovascular mortality by 13% (HR: 0.87; 95% CI: 0.78-0.96) and nonfatal myocardial infarction (MI) by 9% (HR: 0.91; 95% CI: 0.81-1.01). Researchers observed that GLP-1 RAs had a significantly higher benefit over insulin for all-cause mortality, with an HR of 0.31 (95% CI: 0.20-0.48, I² = 69%). The propensity of insulin to contribute to dyslipidemia, atherosclerosis, hypertension, heart failure, and arrhythmia may lower the safety profile of insulin [25]. Giugliano et al. reported a significant reduction in all-cause mortality in the three major CVOTs: LEADER with liraglutide, EXSCEL with exenatide (Exenatide Study of Cardiovascular Event Lowering), and PIONEER 6 with semaglutide (Peptide Innovation for Early Diabetes Treatment). They found that GLP-1 RAs reduced all-cause mortality by 12% (HR: 0.88; 95% CI: 0.80-0.96; P = 0.012). Additionally, GLP-1 RAs significantly reduced the risk of hospitalization for heart failure by 10% (HR: 0.90; 95% CI: 0.82-0.99; P = 0.023) and provided robust benefits in reducing the incidence of macroalbuminuria by 26% (HR: 0.74; 95% CI: 0.67-0.82; P < 0.001) [24]. These findings emphasize the potential of GLP-1 RAs as a preferred treatment option for type 2 diabetic patients who are at high risk of cardiovascular events.

Cardiovascular Risk Factors

Qin et al. undertook a meta-analysis on the major CVOTs, including the EXSCEL trial, LEADER study (liraglutide), SUSTAIN 6 study (semaglutide), Harmony Outcomes study (albiglutide), ELIXA study (Evaluation of Lixisenatide in Acute Coronary Syndrome), and REWIND study (dulaglutide). They observed that no studies investigated the differences between the various classes of GLPs and their cardio-protective profiles and that their effects might differ based on their structure or potency. Improvements in cardiovascular risk variables such as HbA1c, systolic blood pressure, body weight, and anti-inflammatory pathways may explain the cardioprotective effects of GLPs. Specifically, GLP-1 RAs reduced the risk of death from cardiovascular causes by 10% (RR: 0.90; 95% CI: 0.83-0.97; P = 0.004) and reduced the risk of stroke (fatal or nonfatal) by 15% (RR: 0.85; 95% CI: 0.77-0.94; P = 0.001). GLP-1 receptors in cardiac and vascular tissues also improve endothelial function, cardiac output, and myocardial glucose uptake [20]. Tuttolomondo et al. also reinforced these findings. They observed that patients treated with dulaglutide showed significantly better metabolic profiles, with a 26% reduction in macroalbuminuria (HR: 0.74; 95% CI: 0.67-0.82; P < 0.001), which was associated with positive findings on vascular health markers like arterial stiffness and endothelial function markers [30]. Zhang et al. also confirmed these findings by demonstrating that treatment with GLPs reduced the carotid intima-media thickness (CIMT) significantly by -0.14 mm (95% CI: -0.25, -0.02; P = 0.016) compared to insulin therapy, which correlated to reduced low-density lipoprotein and eventually delayed the development of atherosclerotic cardiovascular disease [31].

Challenges in Prescription

Scheen et al. observed that the clinical use of GLPs remains low even though there is increasing evidence regarding the efficacy of these novel agents in reducing cardiovascular complications. Prescriptions for medications like sulfonylurea, DPP4is, and insulin continue to increase [18]. Reluctance from the side of prescribers and patients, gastrointestinal adverse effects, the high price of the medication, and difficulties in navigating the healthcare system are some of the reasons for this trend. Huang et al. in their study observed that the liraglutide group had reduced median per-patient per-month (PPPM) inpatient as well as emergency room, and total medical expenditures when compared to the baseline insulin group but higher median PPPM outpatient, total pharmacy, and total costs (all P < 0.0001). This observation led to the inference that patients on GLPs had higher pharmacy costs but lower medical costs when compared to insulin users, which, in effect, decreased overall medical expenditure [29]. Available data from studies have demonstrated that improved clinical outcomes balance the higher cost. Efforts should be made at multiple levels, from clinicians to pharmaceutical companies to payers to patients, to ensure the affordability of the medication.

Strengths and limitations

This systematic review included real-world, good-quality studies and multinational trials with large sample sizes and long follow-up periods, which helps minimize the risk of unreliable results. Our review comprised only observational studies, systematic reviews, and meta-analyses characterized by a low risk of bias. The benefits of GLP-1 RAs in reducing arrhythmia and atrial fibrillation were not evaluated in any of the included studies. Also, no studies have compared the efficacy of the different classes of GLPs and their cardioprotective profiles. Excluding studies published before 2020, studies published in languages other than English, and articles unavailable as full-text could be a few of the other limitations of the review.

## Conclusions

GLP-1 RAs are associated with a significant reduction in cardiovascular mortality, all-cause mortality, nonfatal MI, and nonfatal stroke. Although studies have demonstrated a decreased risk of hospitalization from heart failure, this was a minor protective effect and warranted more studies for definitive evidence. The benefits of GLP-1 RAs were more pronounced in inpatient populations with existing cardiovascular risk factors, and more evidence is needed to ascertain whether these results apply to those without any cardiovascular risk factors. Also, more studies are needed to compare the efficacy of the different classes of GLPs and their cardioprotective profiles. A multilevel approach to devise and execute policies is needed to overcome prescription barriers and to ensure optimal usage of these drugs in eligible populations.
